# Pediatric rheumatology education: the virtual frontier a review

**DOI:** 10.1186/s12969-024-00978-0

**Published:** 2024-06-05

**Authors:** Jeanine McColl, Oscar Mwizerwa, Christiaan Scott, Shirley ML Tse, Helen E. Foster

**Affiliations:** 1 Department of Pediatrics, Division of Rheumatology, University of Calgary, Calgary, AB Canada; 2 Department of Pediatrics, Division of Rheumatology, University of Toronto, The Hospital for Sick Children, 170 Elizabeth St, M5G 1E8 Toronto, ON Canada; 3 Clinical Research Centre, University of Cape Town. Red Cross War Memorial Children’s Hospital, Anzio Road Observatory, 7700 Cape Town, Western Cape South Africa; 4 Population and Health Institute, Newcastle University, Framlington Place, NE1 7RU Newcastle upon Tyne, Tyne and Wear UK; 5 Department of Pediatrics, Division of Rheumatology, University of Calgary, Alberta Children’s Hospital, 28 Oki Drive NW, T3B 6A8 Calgary, AB Canada

**Keywords:** Access, Pediatric, Rheumatology, Virtual, Education, Underserviced, Global, Health, Children, Musculoskeletal

## Abstract

**Background:**

Many children with rheumatic and musculoskeletal diseases are unrecognized. Identifying these children requires health care provider awareness, knowledge, and skills to recognize disease features and how (and when) to refer to specialist care. The aim of this paper is to highlight the need for better access to health care, review the essential role that education and virtual care play to address unmet need in low resource areas and especially to expand workforce capacity. Using collaborative partnerships, virtual platforms, and innovative assessment methods, musculoskeletal care and education can be delivered to reach a greater audience than ever before. Increased awareness through multiple initiatives and readily available resources are imperative to improve global rheumatology care.

**Conclusion:**

The needs of children with rheumatic diseases and musculoskeletal conditions are vastly underserved around the world resulting in preventable morbidity and mortality. Expanded implementation of virtual education and e-health care platforms provides an opportunity to increase access to care for children globally.

## Background

There are 6–7 million children globally living with rheumatic diseases, with a large majority of these children residing in low resource income countries [1–4]. The true prevalence of disease is likely underestimated given the lack of epidemiological data and the burden of other conditions including malnutrition and infectious communicable diseases often take precedence in health care resource priority over chronic musculoskeletal (MSK) conditions [5]. Children with rheumatic conditions go unrecognized for many reasons including greater focus on other health care burdens in low-income countries and being lost in the ‘abyss of the unmet global MSK health care need.’ Despite there being effective treatments available, there is often marked delay to appropriate care resulting in worse clinical and socioeconomic outcomes, irreversible joint damage, lifelong disability, lower quality of life, and increased mortality [2, 6–8]. Children with Juvenile Idiopathic Arthritis (JIA) living in countries with lower Gross Domestic Product (GDP) have greater disease activity and damage than those living in wealthier countries [7]. Joint damage is known to be associated with referral delay [7]. *This is a silent global crisis.*

Identifying children with rheumatic disease and MSK disease requires health care providers in community settings to have awareness, knowledge, and skills to recognize the features and know when and how to refer to appropriate specialist providers. The aim of this paper is to highlight education and virtual care can address unmet health care needs in underserviced areas.

## Main text

### Workforce capacity

The global workforce of pediatric rheumatologists is limited with the burden of rheumatic disease far exceeding health care capacity and resources regardless of location and the country’s wealth [7, 8]. Europe has one pediatric rheumatologist for every million children with a higher number of pediatric rheumatologists in Western than Eastern Europe [8]. In order to provide adequate care, it has been established that one pediatric rheumatologist is required for every 200 000 children and at minimum one for every 400 000 children [8–10]. The United States currently meets 64% of the demand with many states not having a pediatric rheumatologist at all [8]. There is a limited physician workforce with 2.7 physicians in continental Africa per 10 000 people compared to 32.1 in Europe [1]. Access to pediatric rheumatology multidisciplinary teams in Africa is scarce. There are challenges defining who qualifies as a pediatric rheumatologist in low resource settings, with various types of practitioners seeing children with rheumatic diseases. It is estimated there are currently 18 trained pediatric rheumatologists in Sub-Saharan Africa. The World Bank estimates Sub-Saharan Africa has 500 million children under the age of 14 which results in an unbelievable ratio of 41.6 million children per pediatric rheumatologist [11]. Health care is often concentrated in large urban areas forcing those living in rural and remote settings to seek care in urban centers, this results in time and transportation costs to families and is often a barrier to care. Given the lack of pediatric rheumatologists, health care systems rely on community health workers, general practitioners, pediatricians, orthopedic surgeons, and adult rheumatologists to identify and manage rheumatic diseases when there is lack of access to specialized care; many have had little training in pediatric rheumatology. Not only is there a lack of physicians but there is a lack of allied health providers (AHP) and multidisciplinary teams in low resource income countries (LRIC) to provide essential care to these children.

### Virtual care

Clinical practice in pediatric rheumatology has traditionally involved face to face consultation. The significant global need and lack of trained providers in LRIC has further exacerbated lack of access to specialist care. Telemedicine has been an important development in other specialties (e.g. radiology/dermatology) but the use of telemedicine in pediatric rheumatology historically has been low due to physical exam limitations associated with virtual platforms [12, 13]. During the Covid-19 pandemic the utilization of telemedicine increased exponentially as practitioners were forced to rapidly develop alternative ways to provide continuity of care especially for vulnerable patients [14]. Pediatric rheumatologists began conducting virtual visits with considerable success, aided by the development of a variation of pGALS for remote working called video/virtual pGALS (v-pGALS) [14–16]. The v-pGALS has been validated with a sensitivity of 93.7% and specificity of 100% for detecting MSK abnormalities [16]. The Childhood Arthritis and Rheumatology Research Alliance (CARRA) reported the MSK exam was the most reliable component of the telemedicine exam and thus reassuring that telemedicine has an important place in clinical care [17]. However, depending on the location, practice licencing can be a challenge as in some countries physicians cannot legally provide virtual care without having a medical license in the country where the patient is located. Given the impact of Covid-19 many governments have reduced licensing restrictions for virtual care [18]. 

Working remotely has normalized virtual care more than ever before. Organizations including the Canadian Rheumatology Association have established a Consensus Statement on best practice for virtual care and developed education modules for providers [19]. There are limitations to virtual care, including provider technology skills, training, and costs associated with equipment set up and maintenance, although ultimately it may prove to be more cost effective by decreasing health care utilization by patients [14]. Advancements in virtual care can be applied to other contexts outside of providing routine care, including increasing access to care in remote and underserviced areas. E-consults provide opportunity for practitioners to obtain advice on cases from experts remotely as well as being a valuable tool for training. Ensuring that patient confidentially is maintained by performing virtual visits in an appropriate area such as clinic room or office with the door closed and ensuring electronic information is secured in accordance with organization policies is required. Patients should be made aware of the limits of confidentiality when using a virtual platform. Benefits to patients by using virtual platforms include limited or no travel required, decreased travel costs, increased convenience, and increased family members in attendance at appointments. Telemedicine models of care in Chile have demonstrated decreased absenteeism from school, decreased pain and immobility from travel time to appointments, and decreased food costs. Patients were able to have an increased number of appointments, increased disease knowledge, medication adherence, and earlier access to biologic therapy [20]. 

Project Extension for Community Healthcare Outcomes (ECHO) is an education model which aims to increase access to evidence-based guidelines and enhanced patient care in underserved communities [21]. It provides participants the opportunity to present anonymized real cases to specialists for discussion and uses technology to leverage resources and provides a setting for learning, knowledge translation, and refinement with mentoring and peer support. It has multiple programs dedicated to bone health and rheumatology. This model has great potential and has been shown to improve patient outcomes and ultimately improve community health [21]. There is however an associated fee which may be a barrier for health care providers and communities.

Telehealth provides an avenue to extend knowledge translation and training opportunities to under serviced areas. The Covid − 19 pandemic saw the implementation of virtual and hybrid scientific meetings. This provides increased flexibility and access for practitioners who may not previously have had time, funding, travel, or visa limitations to attend in person conferences. Advantages of online learning over traditional models of instruction include the ability to update material in a timely manner [22]. This ensures learners are obtaining the latest evidence-based content. E-learning has been demonstrated to be as effective as conventional methods, is convenient, and can be used to foster self-directed learning [22]. Literature has shown the main barriers which affect creation and implementation of online medical education materials include poor technical skills, lack of time, and inadequate infrastructure in addition to absence of organization strategies [23]. These can be overcome by improved educator technology skills, incentives, institutional support, and strategies to support online education [23]. 

**Education**

In LRICs pediatric rheumatic diseases are given little attention in training, health care planning, and budgeting which likely results in decreased diagnosis and referrals to appropriate providers [10]. Lack of training and exposure to MSK conditions during medical training results in few primary care providers and pediatricians recognizing and diagnosing them. Where there is education regarding MSK conditions, it is often adult or orthopedic oriented leaving a substantial gap in knowledge about pediatric rheumatic conditions [24]. In Africa, for example, there are limited pediatric rheumatology training centers with only three formal programs in South Africa (University of Cape Town, Stellenbosch University, and University of the Witwatersrand).

To address the need for care while managing limitations of the current workforce capacity, increasing MSK and pediatric rheumatology education through training models of care have been implemented. During the Covid-19 pandemic multiple free webinars have arisen through the Pediatric Society of the African League Against Rheumatism (PAFLAR) and Asia Pacific League of Associations for Rheumatology (APLAR) [4, 25].

In the past PAFLAR has collaborated with the Juvenile Inflammatory Rheumatism (JIR) Winter School from Switzerland to provide webinars and courses for those providing care to children with rheumatic disease [1]. This project was funded by the International League Against Rheumatism (ILAR).

There are structured training programs including the UWEZO (meaning “capability” in Swahili) project which is a collaborative effort between Kenyan, Swedish, and United Kingdom rheumatologists, researchers, and patients that is increasing access to MSK health care providers through a sustainable training program at 11 locations in Kenya [26]. The Enhancement of Pediatric and Adult Rheumatology Education and Practice Project (EPAREP) was supported by ILAR with a goal to improve the management and care of patients with rheumatic disease through enhanced rheumatology education and advocacy in Zambia [1, 27]. 

Education initiatives have been developed by “Rheumatology for All” which works primarily in Ethiopia and Rwanda; their mission is to increase access to rheumatology care in under resourced regions by funding education of local physicians to become rheumatologists and provide education for local physicians [1, 28]. “The East African Initiative” was developed by ILAR and provided training for the two pediatric rheumatologists in Kenya [29]. The Pediatric Rheumatology European Society Rheumatologists and Researchers (PReS EMERGE) offers fellowship programs where recipients obtain a bursary to attend a European rheumatology center for six monthsand this has been accessed by trainees from LRIC to gain further education, clinical, and research exposure [30]. PReS has the Sisters Hospital Initiative where centers are paired and develop regular contact with educational events done remotely. PReS has Basic Rheumatology Courses which are typically held in LRICs to reach non-specialists in pediatric rheumatology and use a model of regional and international faculty to facilitate networking and collaboration [31].The PReS Basic Courses were originally ‘"face to face” but have moved to a virtual / hybrid platform since the Covid-19 pandemic. The American College of Rheumatology (ACR) and APLAR have a clinical and research exchange program that allows participants to share knowledge, experience, and fosters collaboration opportunities [32]. 

APLAR developed the “Asia-Pacific Initiative for Rheumatology Nurse Education” (ASPIRE) program introducing a nurse specialist role to clinical practice and to address the caseload challenges facingrheumatologists [25]. This program offers nurses education and skills and allows for patient clinical support through assessment, patient education, and care [33]. Another approach is a shared model, with in person training at local and international centres which are available in Oman, Kuwait, and Saudi Arabia. This provides opportunity to learn standards of care, networking, and practices established elsewhere along with the challenge of determining how they can be applied to a local context.

Pediatric musculoskeletal matters (PMM)– www.pmmonline.org/doctor and pediatric musculoskeletal matters-nursing (PMMN)– www.pmmonline.org/nurse are free and open to all, providing evidence based online resources targeting students (medical, nursing and allied health), general pediatricians, family medicine doctors, orthopedics, and community health care workers [15]. PMM includes pGALS (pediatric Gait, Arms, Legs, Spine) with multiple language translations, V-pGALS (video pediatric Gait, Arms, Legs Spine) and interactive e-modules to provide self-directed learning [34–37]. The PMM Editorial Board comprises pediatric MSK experts, allied health, nurses, and parent advocates from around the world to ensure the content is relevant for LRIC settings. As of January 2024 PMM had over 1.2 million hits from over 502 000 users in 224 countries [15]. General pediatrics training programs in Thailand have incorporated PMM and pGALS with a Thai language translation into their four-week pediatric rheumatology curriculum and have demonstrated increased learner confidence in their MSK assessment skills [38]. PMM also includes community MSK triage guidance, information on normal MSK development, when to be concerned, and targets the educational needs of health professionals working in family medicine or community settings [39, 40]. 

### Awareness

There are various organizations working toward increasing access and health care for children with rheumatic and MSK conditions as outlined in Table 1.

**Table 1:** Global initiatives to increase awareness about rheumatic and MSK diseases

Organization	Details
The Pediatric Global MSK Task Force	Instrumental in the promotion of bone and joint health, sharing models of good practice to address the gap in care, and advocating for increased pediatric MSK resource allocation [3].
World Arthritis Day	Established by the Arthritis and Rheumatism International (ARI) in 1996 and is held annually on October 12th to raise awareness of the existence and impact of rheumatic and MSK diseases [41].
World Young Rheumatic Disease Day (WORD Day)	Is an annual event that occurs on March 18th to promote knowledge and awareness in parents, teachers, primary care providers, physicians, and the public to assist in early identification, diagnosis, and referral to pediatric rheumatology [42].
Juvenile Arthritis Foundation of Australia (JAFA)	In partnership with clinicians and parents JAFA has lobbied the Australian parliament and successfully obtained recommendations regarding workforce, clinical care, research, education, and funding [43].
Tin Soldiers	Aims to find people living with Fibrodysplasia Ossificans Progressiva and other rare musculoskeletal conditions, who currently live without a diagnosis [44]. This initiative aims to identify patients and connect them to a pathway to care.
Miracle Feet	The goal of this organization is to create universal access to treatment for clubfoot and includes a ‘teach the teachers’ program to cascade knowledge to health care professionals working in communities [45].

## Conclusion

Access to care for children with rheumatic diseases and MSK conditions is vastly underserved and results in significant pain, physical, social, functional, and economic disability for many children around the world [3]. It is essential that the global community work together to address the needs of these children who through no fault of their own, but through structural and societal barriers have been subjected to preventable pain, disability, and distress. Timely access to health care is a fundamental human right. 

Developing education and training initiatives that are relevant and accessible to providers in LRIC at all levels is the essential step to addressing this international health crisis. Action requires empowering local health care providers and enabling sharing of knowledge from experts with experience in LRIC to develop sustainable programs and resources that are relevant at the national and community levels. Ideally these programs would comprise practical clinical experience with access to free online resources (e.g. PMM) in combination with existing programs such as the PAFLAR/APLAR open webinar series and access to speciality training initiatives funded by larger organizations (such as PreS and ACR). 

Continued incorporation of virtual education and e-health care platforms into clinical training of the workforce provides opportunity to increase access to care for children living in underserviced areas. To increase education, awareness, and improved care for children with MSK conditions there must be a defined shared vision with tangible goals, standards with measurable outcomes and accountability. This will require investment and strategic collaboration amongst key participants including international organizations, government officials, education institutions, educators, community partners, local health care providers, and community members. Children with rheumatic and MSK conditions are entitled to safe equitable health care and we all can play an important role in ensuring this becomes a reality regardless of where a child was born.

## Resources

There are multiple resources available for practitioners to learn more about MSK and rheumatic diseases in children, these include:

- Pediatric Musculoskeletal Matters (PMM): https://www.pmmonline.org/doctor/.
- Pediatric Gait, Arms, Legs, and Spine (pGALS): https://www.pmmonline.org/doctor/clinical-assessment/examination/.
- Video Pediatric Gait, Arms, Legs, and Spine (v-pGALS): https://www.pmmonline.org/doctor/clinical-assessment/examination/virtual-or-video-pgals-v-pgals/.
- PAFLAR Webinar Series: https://paflar.org/activities/.
- Versus Arthritis Flip book: https://www.flipsnack.com/BC857F5BDC9/versus_arthritis-hcp_handbook-interactive.html.

Curricula:

- Asia Pacific League of Association for Rheumatology https://aplar.org/academy/upcoming-course/ [46].
- American College of Rheumatology: https://rheumatology.org/general-educator-resources [47].
- European Alliance of Associations for Rheumatology: https://esor.eular.org/ [41].
- Canadian Rheumatology Association: http://rheum.ca/education/educational-resources/ [48].
- The Hospital for Sick Children Pediatric Rheumatology Guide: https://renaissance.stonybrookmedicine.edu/sites/default/files/2019_Revised_Residents_Guide__FINAL.pdf [49].
- Word Young Rheumatic Disease Day: www.wordday.org [42].
- PReS Emerge Fellowship: https://www.pres.eu/emerge/fellowship-programs.html [30].
- PReS Sister Hospitals: https://www.pres.eu/education/sister-hospital.html [50].

## Data Availability

Not applicable.

## Abbreviations

MSK: Musculoskeletal
JIA: Juvenile Idiopathic Arthritis
GDP: Gross Domestic Product
AHP: Allied Health Providers
LRIC: Low Resource Income Countries
pGALS: Pediatric Gait Arms Legs Spine
vpGALS: Video/Virtual Pediatric Gait Arms Legs Spine
CARRA: Childhood Arthritis Rheumatology Research Alliance
ECHO: Extension for Community Health Outcomes
PAFLAR: Pediatric Society of the African League Against Rheumatism
APLAR: Asia Pacific League of Associations for Rheumatology
JIR: Juvenile Inflammatory Rheumatism
ILAR: International League Against Rheumatism
EPAREP: Enhancement of Pediatric and Adult Rheumatology Education and Practice Project
PReS: Pediatric Rheumatology European Society
PReS EMERG: Pediatric Rheumatology european Society ‘EMErging RhumatoloGists and Researchers
ACR: American College of Rheumatology
ASPIRE: Asia Pacific Initiative for Rheumatology Nurse Education
PMM: Pediatric Musculoskeletal Matters
PMMN: Pediatric Musculoskeletal Matters Nursing
ARI: Arthritis and Rheumatism International
